# Cross-border movement of older patients: a descriptive study on health service use of Japanese retirees in Thailand

**DOI:** 10.1186/s12992-017-0241-9

**Published:** 2017-03-08

**Authors:** Yumiko Miyashita, Chutima Akaleephan, Nima Asgari-Jirhandeh, Channarong Sungyuth

**Affiliations:** 1 International Health Policy Program, Thailand, Ministry of Public Health, Tiwanon Road, Nonthaburi, 11000 Thailand; 2 World Health Organization, Regional Office for South East Asia, World Health House, Indraprastha Estate, Mahatma Gandhi Marg, New Delhi, 110002 India; 3 Previously with International Health Policy Program, Thailand, Ministry of Public Health, Tiwanon Road, Nonthaburi, 11000 Thailand

**Keywords:** Trade in health services, Long-stay tourism, Retirees, Health service use, Japanese, Thailand

## Abstract

**Background:**

Thailand’s policy to promote long-stay tourism encourages Japanese retirees to relocate to Thailand. One concern of such an influx is the impact of these elderly foreign residents on the Thai health system. This study aims to reveal the current use of and needs for health services amongst Japanese retirees residing in various locations in Thailand.

**Methods:**

In collaboration with nine Japanese self-help clubs in Bangkok, Chiang Mai, Chiang Rai, and Phuket, questionnaire surveys of Japanese long-stay retirees were conducted from January to March 2015. The inclusion criteria were being ≥ 50 years of age and staying in Thailand for ≥30 days in the previous 12 months while the main exclusion criteria included relocation by company, relocation due to marriage, or working migrants.

**Results:**

The mean age of the 237 eligible participants was 68.8, with 79.3% of them being male, 57.8% having stayed in Thailand for ≥5 years, 63.3% having stayed in Thailand for ≥300 days in the previous 12 months and 33% suffering from chronic diseases or sequelae. Of the 143 who had health check-ups in the previous 12 months, 48.3% did so in Thailand. The top 3 diseases treated either in Thailand or Japan in the previous 12 months were dental diseases (50 patients), hypertension (44 patients), and musculoskeletal disorders (41 patients), with the rate of treatment in Thailand standing at 46.0, 47.7, and 65.9%, respectively. Of the 106 who saw a doctor in Thailand in the same period, 70.8% did so less than once a month. Only 23.2% of the participants preferred to receive medical treatment for serious conditions in Thailand. However, this number rose to 32.9% for long-term care (LTC) use.

**Conclusion:**

The usage of Thai health services amongst Japanese long-stay retirees is currently limited as they prefer going back to Japan for health screenings and treatment of chronic or serious diseases. However, the number of Japanese residents requiring health services including LTC and end-of-life care is expected to increase. The potential impact of promoting long-stay tourism on the Thai public health should be acknowledged and investigated by the Thai government, including the tourism authority.

## Background

As in many other countries, Thailand’s health sector, which has been closed and nationally focused, is gradually feeling the impact of globalization with the increasing awareness that health care goods and services provided for foreign patients or firms abroad can be a potential income-generating mechanism for the economy [1]. Substantial growth is seen in trade in health services via “consumption abroad”, which refers to the cross-border movement of patients [2]. Currently, many developing countries attract patients from developed countries as well as neighboring countries by providing high quality and affordable treatment, specialized services, or alternative therapies [3]. This benefits the destination countries via the inward flow of funds into the economy, allowing them to upgrade health care infrastructure and technologies and increase employment of health personnel. Conversely, unless properly managed, this model has the potential to widen the gap between private health care providers focusing on foreign patients and wealthy nationals - who can afford technologically-advanced and high-quality health care - and the rest of the health care providers available to the general public [2–6].

**Fig. 1:**
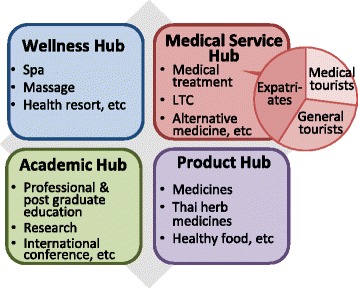
Framework of the Thai medical hub policy. Ref: Ministry of Public Health. Summary of Thailand Health Tourism. Nonthaburi: 2014

Thailand is the largest exporter of health services via consumption abroad in Southeast Asia [6] with 1.4 million foreign patients in 2013 [7], owing to the efforts of private hospitals to develop a new customer base in cooperation with the government’s medical hub policy. The medical hub policy is the government’s overall strategy for ensuring that Thailand becomes medical hub of Asia. It has four components (see Fig. 1) of which Medical Service Hub is one. The Medical Service Hub focuses on three groups of potential foreign patients: i) expatriates living in Thailand; ii) general tourists who will need medical attention while in Thailand; and iii) medical tourists who come specifically for medical reasons.

The largest group of foreign patients are Japanese [6, 7], most of whom are expatriates [8]. Currently there are few medical tourists from Japan since the Japanese universal health insurance scheme allows Japanese to get quality health care in Japan at a moderate price. Thus, this paper focuses on Japanese expatriates only. In 2014, 1.3 million Japanese visited Thailand [9]. The registration data of overseas residents in Thailand indicated that the number of Japanese expatriates had doubled to 64,000 compared to a decade ago [10], thereby making Thailand the country with the fifth-highest Japanese expatiate population [11]. The vast majority of these people were corporate employees and their families who had been transferred from Japan [11]. Notably, there were more than 10,000 older persons aged 60 and above [11], reflecting population aging at home and the promotion of long-stay tourism in both countries.

International long-stay tourism - also known as international retirement migration - became popular in Western countries in the 1960s and has gradually spread globally [12]. The Thai government, particularly the Tourism Authority of Thailand (TAT), has been actively promoting long-stay tourism since 2001 as part of the national development strategy [13]. “Long Stay” is regarded as staying in the country for more than 30 days and not for sightseeing activities or working but with a purpose of living with the intention to return to the home countries [14]. As retirees are the main target group, a special renewable one-year visa is provided for people aged 50 years and above who fulfill certain financial criteria. The Thai government has designated Japan as a primary target country [13, 15].

In Japan, outbound long-stay tourism for leisure was introduced in the 1990s and has been successfully promoted by the private sector [16]. The favored destinations have recently shifted from English-speaking developed countries such as US (particularly Hawaii), Canada, and Australia to Southeast Asian countries due to the low cost of living, short flight time, warm weather, and availability of a long-term visa [17]. Thailand is Japan’s second-most popular long-stay destination [18]. While the exact number of long-stay retirees in Thailand is unknown due to the variety in types of stays and visas [19, 20], Immigration Bureau statistics suggest that at least 3,000 Japanese stayed in Thailand for over a year using retirement visas in 2014 [21], up from 1,400 in 2007 [22].

Long-stay retirees are closely linked to health services. First, according to a survey by the TAT, one of the main determinants of long-term stay in Thailand for Japanese nationals is the availability of medical services [15]. Secondly, older people require more frequent treatment compared to younger age groups due to a higher prevalence of chronic diseases. Previous surveys conducted in the 2000s revealed that among Japanese adults visiting or living in Thailand, the most common causes for seeking treatment were acute respiratory diseases, acute digestive tract disorders, and infections; chronic diseases such as metabolic disorders or circulatory diseases had lower proportions [8, 23]. However, the volume and pattern of medical service use may be shifting due to the increase in the number of Japanese retirees in recent years. Thirdly, in addition to the pull factor from Thailand, the increasing shortage of care workers and facilities for long-term care (LTC) for the elderly with difficulties in activities of daily living (ADL) in Japan is also convincing Japanese retirees to move abroad [13, 16, 24]. Hence, the growing number of long-stay retirees will increase medical and long-term care demand in Thailand, and thus have an impact on the overall Thai health care system - which already faces a shortage of human resources and has to tackle its own aging population.

Information on actual health care use of long-stay retirees in foreign countries is difficult to come by. Even in Western countries with a history of retirement migration, there are only a small number of studies on health care issues of long-stay retirees, most of which are qualitative studies on their health care experience abroad [25–27]. Previous studies of Japanese retirees in Thailand were mostly conducted in Chiang Mai, a mecca for long-stay Japanese located in the north of Thailand, and focused on their motivations for coming or leaving Thailand [24, 28, 29], how they adjusted to the host country [20], or improvement of information provision [14]. Few studies reported health care service use quantitatively except for a study in 2007 in Chiang Mai which focused on the attitude of Japanese long-stay retirees towards Thai medical services [30]. Apart from the limited data gained from this study in Chiang Mai, very little information is available on health care demand, the frequency of medical service use, or disease patterns of Japanese retirees in Thailand.

Therefore, this study aims to reveal the current use of and needs for health services amongst Japanese long-stay retirees, with a view of providing insight into the impact of long-stay tourism on the Thai health system.

## Methods

A cross-sectional survey was conducted from January to March 2015, coinciding with the high season for Japanese long-stay tourists in Thailand. Convenience sampling methods were used in which study participants were recruited in cooperation with nine Japanese self-help clubs on a voluntary basis from different cities: Bangkok (3), Chiang Mai (3), Chiang Rai (1), and Phuket (2). The questionnaire was self-administered and anonymized. Sampling and data collection methods differed depending on the club’s style of activities and member registration. In five clubs, questionnaires were distributed during periodical meetings and collected at the meetings or posted back. Postal surveys were conducted in the remaining four clubs. In two of the clubs, questionnaires were posted to the members aged 50 and over and collected by post. In another club, questionnaires were handed out at the office and returned by mail. In the final club, some members were handed out questionnaires in the office and returned it by mail while others were interviewed by phone.

The focus of the study was Japanese long-stay retirees, including those who are semi-retired. Inclusion criteria were i) at least 50 years old according to the requirement of the long-stay/retirement visa and ii) staying in Thailand for more than 30 days in the previous 12 months. Exclusion criteria include i) expatriate personnel and their spouses who were relocated to Thailand by Japanese companies; ii) relocation due to marriage; iii) working migrants; iv) new arrivals who started their long-term stay in the past three months; or v) individuals who left more than 30% of the questionnaire blank. Relocation due to marriage was identified by two variables: those who started their long-term stay before reaching 50 years of age and had a Thai spouse. Working migrants were assumed to be those who started their long-term stay in Thailand before reaching 50 years of age and received their primary income from working in Thailand.

The data obtained included i) basic demographics and socio-economic background such as age, sex, marital status, and education level; ii) condition of stay including the number of years living in Thailand as a long-stay visitor, number of days staying in Thailand in the previous 12 months, and living expense per month; iii) health status such as BMI calculated by height and weight, self-reported chronic diseases or sequelae as well as health related QOL obtained by the EuroQol-5D-5 L [31]; iv) use of health services in the previous 12 months, including health check-ups, cancer screenings, LTC services, and doctor’s visits; v) use of medical services in the previous 12 months such as the frequency of visiting doctors, experience and duration of hospital stays, type of diseases, and medical expenses and its sources; and vi) future intention of using health services, i.e. a preferable place for LTC or medical treatment for severe diseases. LTC services herein refer to physical assistance with bathing, toileting, meals, etc. as well as sputum suction or tube feeding. Descriptive statistics were used with dividing the participants into three groups by location: Bangkok, Chiang Mai, and other areas (hereinafter “Other area”).

The study was approved by the ethical committee of the Institute for the Development of Human Research Protections, Thailand. Implied consent was substituted for written consent to assure anonymity of participants by considering their filled and returned questionnaire as consent for participation in the survey; this implied consent principle was explained beforehand. A letter of approval was also obtained from the representative of each Japanese club.

## Results

### Study participants

**Fig. 2:**
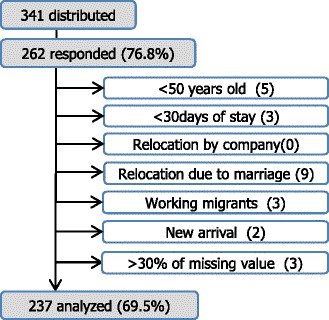
Flow chart of the study participants. Ref: Ministry of Public Health. Summary of Thailand Health Tourism. Nonthaburi: 2014

The questionnaires were distributed to 341 persons, and 262 responded (76.8%). Twenty-five persons were excluded in the following order: less than 50 years old (5); less than 30 days of stay (3); relocation due to marriage (9); working migrants (3); new arrivals within three months (2); and greater than 30% of the questionnaire left unfilled (3). None of the participants were expatriate staff relocated from Japan or their spouses. As a result, data from 237 persons (69.5%) were analyzed as shown in Fig. 2.

### Basic demographics and condition of long stay

Among the eligible study participants living in Bangkok (56), Chiang Mai (144), and Other area (37), 188 were males (79.3%). The mean age was 68.8 years old (SD 5.6) and those aged 75 and more accounted for 16.0%. Retirement visas topped the list for visa status, with 197 persons (83.1%) using them.

A difference was observed in the participants’ socio-economical background and condition of stay based on the area of residence. People married to Japanese or other non-Thais made up the largest group in Bangkok and Chiang Mai (55.4 and 49.3%, respectively), while those married to Thais accounted for 45.9% in Other area. As for living status in Thailand, living alone was most common in Bangkok (46.4%), including 10 persons who were married but not living together in Thailand. In Chiang Mai and Other area, couple households accounted for 47.2 and 48.6%, respectively. Households with a child accounted for a small proportion in Bangkok (7.1%) and Chiang Mai (14.6%) but was commonly observed in Other area (29.7%). Living with a parent was a rare case in all areas, with only four observations in total (1.7%). The percentage of those who had stayed in Thailand for more than a year before the current long-stay status - considered mostly as an expatriate personnel or their family - was higher in Bangkok (33.9%) compared to Chiang Mai (13.9%) and Other area (18.9%). The average monthly household income and expenditure were highest in Bangkok at JPY 300,867 (USD 2,844 1) and THB 61,660 (USD 1,898 2), followed by Chiang Mai with JPY 265,300 (USD 2,508) and THB 50,282 (USD 1,548), and lowest in Other area with JPY 194,400 (USD 1,838) and THB 42,667 (USD 1,314), respectively.

The mean number of years for Long Stay was 6.4 years (SD 4.7). Fifty-nine persons (24.9%) had been staying for more than 10 years as a long-stay visitor, and the rate rose to 35.1% among the respondents in Other area. The median days (1st quartile(Q1)-3rd quartile(Q3)) of stay in Thailand in the previous 12 months was 300 (200–335) days in Bangkok, 320 (270–350) days in Chiang Mai, and 330 (300–360) days in Other area. During the previous 12 months, the most common number of trips to Japan was twice and above (44.7%), and this was particularly higher among Bangkok residents (66.1%). On the other hand, 20.3% did not go back to Japan in the previous 12 months and the Other area rated higher than other cities (29.7%). The percentage of those who kept residence in Japan was 80.4% in Bangkok, 75.0% in Chiang Mai, and 51.4% in Other area. Those who kept residential registration in Japan – a requirement for one to be covered by the Japanese national health insurance (Kokuho) - was 57.1% in Bangkok, 44.4% in Chiang Mai, and 29.7% in Other area (Table 1).

**Table 1:** Basic characteristics and condition of Long Stay

	Bangkok	(*n* = 56)	Chiang Mai	(*n* = 144)	Other	(*n* = 37)	Total	(*n* = 237)
	n	%	n	%	n	%	n	%
Sex								
Male	45	80.4	110	76.4	33	89.2	188	79.3
Female	11	19.6	34	23.6	4	10.8	49	20.7
Age								
Mean (SD)	69.1	(5.6)	68.3	(5.7)	70.3	(4.8)	68.8	(5.6)
Visa type:								
Retirement	47	83.9	121	84.0	29	78.4	197	83.1
Other	7	12.5	20	13.9	6	16.2	33	13.9
Marital status								
Single, divorced or bereaved	20	35.7	32	22.2	5	13.5	57	24.1
Married to non-Thai	31	55.4	71	49.3	15	40.5	117	49.4
Married to Thai	5	8.9	39	27.1	17	45.9	61	25.7
Living status								
Alone	26	46.4	41	28.5	7	18.9	74	31.2
With partner only	21	37.5	68	47.2	18	48.6	107	45.1
Other	7	12.5	30	20.8	12	32.4	49	20.7
Lived in Thailand for >1 year before Long Stay status								
Yes	19	33.9	20	13.9	7	18.9	46	19.4
No	35	62.5	122	84.7	30	81.1	187	78.9
Monthly household income (USD)								
Mean (SD)	2844	(1306)	2508	(1384)	1838	(873)	2478	(1329)
Monthly household living expenditure (USD)								
Mean (SD)	1898	(779)	1548	(643)	1314	(612)	1590	(695)
Years of Long-stay in Thailand								
Mean (SD)	6.4	(5.2)	5.9	(4.2)	8.3	(5.4)	6.4	(4.7)
Days of stay in Thailand in the previous 12 months								
Median (Q1-Q3)	300	(200–335)	320	(270–350)	330	(300–360)	300	(270–350)
Number of trips to Japan in the previous 12 months								
0	4	7.1	33	22.9	11	29.7	48	20.3
1	13	23.2	49	34.0	14	37.8	76	32.1
≥ 2	37	66.1	59	41.0	10	27.0	106	44.7
Keeping a home in Japan								
Yes	45	80.4	108	75.0	19	51.4	172	72.6
No	10	17.9	36	25.0	18	48.6	64	27.0
Residential registration in Japan								
Registered	32	57.1	64	44.4	11	29.7	107	45.1
Removed	23	41.1	78	54.2	26	70.3	127	53.6

### Health status and health service use in the previous 12 months

Most of the participants were found to be relatively healthy. Table 2 shows that the majority (63.7%) were within the normal BMI range (18.5-24.9), and eight persons (3.4%) were underweight (BMI less than 18.5). The rate of overweight (BMI equal to or greater than 25) was lower in Other area (13.5%) compared to Chiang Mai (38.2%) and Bangkok (30.4%). There were 79 persons (33.3%) who had chronic diseases or sequelae from diseases or injuries. As for health-related QOL, the mean EQ-5D Index Score was 0.91 out of 1.0.

**Table 2:** Health status and health service use in the previous 12 months

	Bangkok	(*n* = 56)	Chiang Mai	(*n* = 144)	Other	(*n* = 37)	Total	(*n* = 237)
	n	%	n	%	n	%	n	%
BMI								
< 18.5	3	5.4	4	2.8	1	2.7	8	3.4
18.5-24.9	36	64.3	84	58.3	31	83.8	151	63.7
≥ 25	17	30.4	55	38.2	5	13.5	77	32.5
Chronic disease or sequelae								
Have	19	33.9	43	29.9	17	45.9	79	33.3
Don’t have	36	64.3	78	54.2	19	51.4	133	56.1
QOL; EQ-5D-5 L index value								
Mean (SD)	0.93	(0.11)	0.91	(0.13)	0.89	(0.14)	0.91	(0.13)
Rehabilitation in Thailand								
Received	1	1.8	9	6.3	1	2.7	11	4.6
Not received	55	98.2	131	91.0	34	91.9	220	92.8
LTC services in Thailand								
Received								
At Home	0	0.0	2	1.4	1	2.7	3	1.3
Nursing home/residential care	0	0.0	0	0.0	0	0.0	0	0.0
Elderly nursing unit in hospital	0	0.0	0	0.0	1	2.7	1	0.4
Unspecified place	0	0.0	1	0.7	0	0.0	1	0.4
Not received	46	82.1	123	85.4	31	83.8	200	84.4
Health check-ups								
Thailand	10	17.9	36	25.0	4	10.8	50	21.1
Japan	21	37.5	44	30.6	9	24.3	74	31.2
Both countries	3	5.4	12	8.3	4	10.8	19	8.0
Not received	20	35.7	48	33.3	18	48.6	86	36.3
Cancer screenings (Average: Stomach, Lung, and Colon)								
Thailand	6	10.7	13	9.0	2	6.3	21	9.0
Japan	14	24.4	44	30.6	7	18.0	64	27.1
Both countries	1	2.4	3	2.3	2	4.5	6	2.7
Not received	34	60.1	79	54.9	25	66.7	137	57.9
Consulting a doctor								
Thailand	15	26.8	34	23.6	17	45.9	66	27.8
Japan	10	17.9	26	18.1	5	13.5	41	17.3
Both countries	10	17.9	25	17.4	5	13.5	40	16.9
Not received	16	28.6	53	36.8	10	27.0	79	33.3

Rehabilitation and LTC service use in Thailand in the previous 12 months was small. Eleven persons (4.6%) received rehabilitation and five used LTC services. Only one (0.4%) used LTC services for himself at an elderly nursing unit in hospital, while the remaining four participants (1.7%) used LTC services for a disabled family member, three of whom used at-home services. None of the participants used residential care services.

Interesting health seeking behaviors of Japanese retirees were observed. Of the 143 respondents (60.3%) who received health check-ups in the previous 12 months, most received it in Japan rather than in Thailand. The responses showed that 50 persons (35.0%) received check-ups in Thailand, 74 (51.7%) in Japan, and 19 (13.3%) in both countries. The number and rate of retirees who received screenings for stomach, lung, and colon cancers were 89 (37.6%), 87 (36.7%), and 100 (42.2%), respectively. On average, the vast majority of them received cancer screenings in Japan, comprising 69.9% in Japan, 23.2% in Thailand, and 6.9% in both countries. However, of the 147 (62.0%) who saw a doctor for treatment in the previous 12 months, many people did so in Thailand, comprising 66 persons (44.9%) in Thailand, 41 (27.9%) in Japan, and 40 (27.2%) in both countries.

**Fig. 3:**
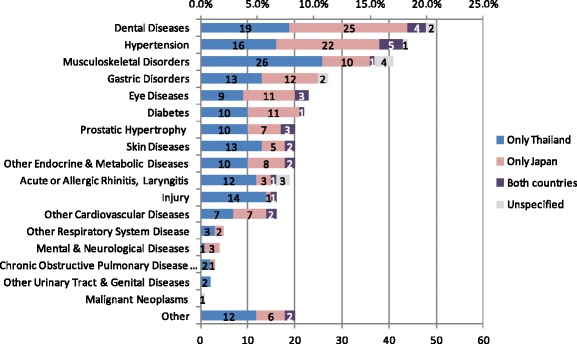
Diseases treated in the previous 12 months. Ref: Ministry of Public Health. Summary of Thailand Health Tourism. Nonthaburi: 2014

As shown in Fig. 3, a large number of people were treated either in Thailand or Japan in the previous 12 months for dental diseases (50 cases; 21.1%), hypertension (44 cases; 18.6%), musculoskeletal disorders (41 cases; 17.3%), gastric disorders (27 cases; 11.4%), eye diseases (23 cases; 9.7%) and diabetes (22 cases; 9.3%). Half of the patients received treatment only in Japan for dental diseases (50.0%), hypertension (50.0%), eye diseases (47.8%), and diabetes (50.0%). On the other hand, disease groups with a high rate of treatment in Thailand were musculoskeletal disorders (65.9%); skin diseases (75.0%); prostatic hypertrophy (65.0%); acute or allergic rhinitis and laryngitis (68.4%); and injury (93.8%). Only a few cases of cerebrovascular diseases (3) and ischemic heart diseases (3) were treated in Thailand. None of the participants were treated for mental diseases or malignant neoplasm in Thailand.

Among the 106 participants (44.7%) who saw a doctor in Thailand in the previous 12 months (Table 3), 93 used private hospitals or clinics (87.7%) while 25.4 and 22.7% of residents in Chiang Mai and Other area used public health facilities, respectively. Twenty-eight samples were observed to have visited medical facilities at least once a month (26.4%). Twenty-three participants were admitted to a hospital (21.7%) but 18 of them were discharged within a week (78.3%) - including 12 within three days (52.2%).

**Table 3:** Use of medical services in Thailand in the previous 12 months

	Bangkok	(*n* = 25)	Chiang Mai	(*n* = 59)	Other	(*n* = 22)	Total	(*n* = 106)
	n	%	n	%	n	%	n	%
Type of hospital/clinics (Multiple answer)								
Private	24	96.0	52	88.1	17	77.3	93	87.7
Public	4	16.0	15	25.4	5	22.7	24	22.6
Frequency of doctor visit								
At least once a month	7	28.0	16	27.1	5	22.7	28	26.4
Less than once a month	18	72.0	40	67.8	17	77.3	75	70.8
Admission to hospitals								
Admitted	7	28.0	13	22.0	3	13.6	23	21.7
Not admitted	18	72.0	41	69.5	17	77.3	76	71.7
Length of stay (days) (*n* = 23)								
Median (Q1-Q3)	3	(2–5)	4	(2–17.5)	3	(3–4)	3	(2–6)

Table 4 shows that 31 respondents paid their entire medical costs out-of-pocket (29.2%). Traveler’s insurance was commonly used by retirees in Bangkok (56.0%) and Chiang Mai (45.8%), while none in Other area used it. The use of Japanese public health insurance accounted for 24.0% of the sample in Bangkok, 22.0% in Chiang Mai, and 13.6% in Other area. The median (Q1-Q3) out-of-pocket medical costs in the previous 12 months was THB 5,000 (0–27,500) or USD 154 (0–847). Only 12 persons spent THB 50,000 (USD 1,539) or more (11.3%) and the maximum amount was THB 200,000 (USD 6,158).

**Table 4:** Medical expenses in Thailand in the previous 12 months

	Bangkok	(*n* = 25)	Chiang Mai	(*n* = 59)	Other	(*n* = 22)	Total	(*n* = 106)
	n	%	n	%	n	%	n	%
Insurance use								
Didn’t use	7	28.0	13	22.0	11	50.0	31	29.2
Used (multiple answer for type)								
Travelers’ insurance	14	56.0	27	45.8	0	0.0	41	38.7
Japanese public health insurance	6	24.0	13	22.0	3	13.6	22	20.8
Private medical insurance	0	0.0	5	8.5	5	22.7	10	9.4
Thai public medical insurance/benefit	0	0.0	0	0.0	3	13.6	3	2.8
Total out-of-pocket medical expense (USD)								
Median (Q1-Q3)	62	(0–770)	216	(46–924)	154	(0–616)	154	(0–847)

### Future intention of use of health services

The majority of participants intended to receive medical treatment in Japan in case of serious diseases (66.2%). Those who preferred Thailand were 3 persons in Bangkok (5.4%), 39 in Chiang Mai (27.1%), and 13 in Other area (35.1%). The number of participants who intended to receive LTC in Thailand in case they became severely disabled was 7 in Bangkok (12.5%), 51 in Chiang Mai (35.4%), and 20 in Other area (54.1%). Among them, home care was the most preferred option (61.5%) - especially true in Other area (85.0%) - followed by care at nursing homes (17.9%) (Table 5).

**Table 5:** Future intention of health service use

	Bangkok	(*n* = 56)	Chiang Mai	(*n* = 144)	Other	(*n* = 37)	Total	(*n* = 237)
	n	%	n	%	n	%	n	%
Preferred place of treatment when one has serious disease								
Japan	44	78.6	91	63.2	22	59.5	157	66.2
Thailand	3	5.4	39	27.1	13	35.1	55	23.2
Don’t know, other, either	4	7.1	11	7.6	1	2.7	16	6.8
Preferred place of LTC when one become severely disabled								
Japan	36	64.3	82	56.9	11	29.7	129	54.4
Thailand	7	12.5	51	35.4	20	54.1	78	32.9
Don’t know, other, either	8	14.3	9	6.3	6	16.2	23	9.7
Type of LTC in Thailand^a^ (Multiple answer)								
At home	4	57.1	27	52.9	17	85.0	48	61.5
Elderly nursing home	2	28.6	11	21.6	1	5.0	14	17.9
Elderly nursing unit in hospital	1	14.3	5	9.8	3	15.0	9	11.5
Don’t know, other	3	42.9	11	21.6	0	0.0	14	17.9

^a^Denominator in the calculation of percentage is those who answered “Thailand” as preferred place of LTC

## Discussions

The study suggests rapid aging among Japanese long-stay retirees with an increase from an average of 64.7 years old in a previous survey in Chiang Mai in 2007 [30] to 68.3 years old in Chiang Mai or 68.8 years old among the total participants. One reason may be that the original cohort continued to stay in Thailand while the other may be that new entrants are older due to an increase in the retirement age or the introduction of a post-retirement employment system in Japan [32].

After more than a decade of Thai long-stay tourism promotion, many Japanese retirees have rooted themselves in Thailand. The majority in the current study (58%) have been staying in Thailand for more than five years, while only 12% did so in 2007 [30]. In addition, a majority of the participants (63%) resided in Thailand for more than 300 days in the previous year. This is understandable since 40% of our participants have either Thai spouses or do not keep a residence in Japan.

Retirees' use of health services in Thailand is infrequent and primarily for non-serious conditions. Many retirees were observed to have utilized preventive services in Japan more than Thailand with 52% versus 35% for health checkups, respectively and 70% versus 23% for cancer screenings, respectively. They may feel that such screening services are more accessible in Japan where they are routinely provided by local authorities under the law and user fees are fully or partly subsidized.

Although one-third of the participants had chronic diseases or sequelae, only a quarter of them saw a doctor once a month or more. As expected, treatment for chronic diseases in the cohort was more commonly observed than among working-age Japanese living in Thailand [8, 23]. However, half of the people who saw a doctor for indicator diseases such as hypertension or diabetes in the previous 12 months did so only in Japan. One possible reason is that patients do not need a doctor’s prescription to purchase their medication from private pharmacies in Thailand. Thus, there was no need to visit a doctor unless the disease’s condition became worse 3.

Unlike medical tourists, a majority of retirees intend to go to Japan for treatment of serious diseases. This was particularly high for the Bangkok residents where nearly 80% indicated this option even though Bangkok holds many excellent hospitals providing advanced medical technology and Japanese-speaking doctors or translators. Although it requires further study, one of the biggest barriers to seeking treatment in Thailand might be a fear of high medical costs. Compared to the fixed medical fees under Japan’s universal health insurance scheme, private medical care costs in Thailand are more expensive [30, 33].

Many of the Japanese retirees living in Thailand have no health insurance. In our study, of those who used health services, one-third paid their medical expenses fully out-of-pocket. It can be reasoned that this does not only stem from individual choice but also from system restrictions. More than half of the participants had withdrawn from the Japanese national health insurance either by choice or by becoming ineligible since they were no longer residents in Japan. Additionally, traveler’s insurance is duration specific; generally valid for a maximum of one year from initiation of travel from Japan. At least 20% of the participants were assumed to be ineligible for traveler’s insurance since they did not go back to Japan within one year. More inconveniently, it is commonly inapplicable for chronic diseases [33] and often excludes the elderly. Private medical insurance is not available for many retirees since it imposes an age limit or medical exclusions. Thus, when faced with high medical costs, many retirees typically decide to terminate their long-stay status in Thailand and return to Japan as the individual co-payment is less [28].

The study suggests that Japanese long-stay retirees are relatively healthy since those who need continuing care for chronic or serious diseases do not tend to remain in Thailand. However, unlike working expatriates who are taken care of through occupational health or private insurance schemes, long-stay retirees have to rely on themselves. It is important to promote their access to quality health care services including preventive care in Thailand.

As for LTC, only five cases utilized LTC services in Thailand as either a care recipient or a family carer, suggesting that retiring to Thailand for LTC usage is not yet common. However, many retirees, particularly those living outside of Bangkok, were staying in Thailand in anticipation of using LTC services in the future even though they cannot use Japan’s public LTC insurance abroad. The desire for LTC has existed for some time [30] and for many of those living in Thailand over many years, this might not be a vague desire any longer but a realistic option instead. In fact, Japanese retirees established the “Society for the Study of Care and Support in Chiang Mai” in 2014 to develop the LTC environment there [28]. Among those indicating use of LTC in Thailand, around 60% desired home care; the majority of these responses have a Thai spouse. Since it is expected that most of them will be cared for by their wives or children and/or live-in maids [28], whose round-the-clock LTC service is much cheaper in Thailand than in Japan, demand for trained LTC workers seems to not be very high. However, the answers were based on the current situation in which few quality nursing homes are available at an affordable price [28]. It remains possible that an increase in the supply of appropriate nursing homes will induce demand from current long-stay retirees as well as the growing numbers of so-called “LTC refugees” from Japan, where more than half a million of older adults are on a waiting list for admission to nursing homes under the public LTC insurance system [34, 35].

Though the TAT initially defined long-stay tourists as people who go eventually back to their home country, the real situation is changing in the mature phase of long-stay tourism since the one-year retirement visa is extendable as often as needed so long as one fulfills certain criteria. The percentage of retirees who intends to receive LTC in Thailand is not high but the number is increasing. Whereas previous studies showed that the impact of medical tourists on the Thai health system is marginal [1, 36], unlike them, older permanent residents will require medical and nursing care for a long period of time and eventually end-of-life care as well. Once they become frail, they cannot easily return to Japan to seek health services as is currently observed. In addition, many of those who currently have private medical insurance will become ineligible as previously discussed. Authorities in both Thailand and Japan should realize the situation and prepare for the growing need of health services including LTC and end-of-life care.

### Limitation

We recruited participants through Japanese self-help clubs but the majority of long-stay retirees do not actually interact with these clubs since many of them come to Thailand for 1–2 months a year only. As such, our sample is biased towards long-stay retirees mainly living in Thailand. Half of the participants were recruited at club meetings, indicating that they are healthy enough to attend. This selection bias may underestimate medical service usage amongst the long-stay retirees mainly living in Thailand.

In addition, telephone interviews (42 cases) might generate information bias. However, the interviews were done by reading out the questionnaire, in which most of the questions including those about health service use were simple questions on objective facts with dichotomous or multiple options without socially desirable answers. Thus, it was unlikely to cause confusion or hesitation for both questioning and answering. The statistical test between interviewees and self-administered respondents in the same club showed no ostentatious or desirable answers by interviewees. The only matter for concern is that 40% of the interviewees did not answer whether they had chronic diseases or not, possibly due to the technical error by the interviewer. However, this bias does not militate against our main findings, i.e. infrequent use of health service use in Thailand even among those with chronic diseases.

There is a possibility of recall bias but it is considered to have little effect on our main findings since they might not forget the experience of serious diseases or continuing care.

## Conclusion

This is the first research on health service usage of Japanese long-stay retirees conducted in various locations in Thailand. The usage of health services amongst Japanese long-stay retirees was found to be currently limited in all study areas due to retirees’ preference for Japan over Thailand for health screenings and treatment of chronic or serious diseases. However, the results suggest that the number of older permanent residents who will require health services including LTC and end-of-life care will increase, especially outside of Bangkok. Ensuring access to quality health care is crucial for the promotion of long-stay tourism since it is a decisive factor when individuals choose or terminate long-stay residence.

The potential impact of long-stay tourism promotion on the Thai public health should be acknowledged and investigated by the Thai government, including the tourism authority. Considering that cross-border health seeking behavior depends on the health care system and environment of one’s country of origin, further research should focus on retirees from other countries.

## Footnotes

1 Calculated by 1USD = 105.79 JPY using mid rate in 2014 at Tokyo Foreign Exchange Market (Ref. Bank of Japan. Reference - Transaction condition at Tokyo Foreign Exchange Market (in 2014). https://www.boj.or.jp/statistics/market/forex/fxdaily/ex2014.htm/. Accessed 11 May 2016)

2 Calculated by 1USD = 32.48 THB using mid rate in 2014 amongst commercial banks in Bangkok Metropolis (Ref. Bank of Thailand. Rates of Exchange of Commercial Banks in Bangkok Metropolis. http://www2.bot.or.th/statistics/ReportPage.aspx?reportID=123&language=eng. Accessed 11 May 2016)

3 Suggested during additional informal group discussion with retirees
