# Erratum to: Cross-border movement of older patients: a descriptive study on health service use of Japanese retirees in Thailand

**DOI:** 10.1186/s12992-017-0247-3

**Published:** 2017-03-29

**Authors:** Yumiko Miyashita, Chutima Akaleephan, Nima Asgari-Jirhandeh, Channarong Sungyuth

**Affiliations:** 1 International Health Policy Program, Ministry of Public Health, Tiwanon Road, Nonthaburi, 11000 Thailand; 2 World Health Organization, Regional Office for South East Asia, World Health House, Indraprastha Estate, Mahatma Gandhi Marg, New Delhi, 110002 India; 3 Previously with International Health Policy Program, Ministry of Public Health, Tiwanon Road, Nonthaburi, 11000 Thailand

## Erratum

In the original publication of this article [1], published on 8 March 2017, figure 2 and 3 contained a wrong figure caption. The original captions are as followed:

- Figure 2 Flow chart of the study participants. Ref: Ministry of Public Health. Summary of Thailand Health Tourism. Nonthaburi: 2014
- Figure 3 Diseases treated in the previous 12 months. Ref: Ministry of Public Health. Summary of Thailand Health Tourism. Nonthaburi: 2014

However, these figures are not taken from the reference Nonthaburi 2014, and the captions should have read:

- Figure 2 Flow chart of the study participants.
- Figure 3 Diseases treated in the previous 12 months.

The original publication of this article has been corrected. The publisher apologizes to the readers for the inconvenience caused by this typesetting error.
